# Aromatherapy: composition of the gaseous phase at equilibrium with liquid bergamot essential oil

**DOI:** 10.1186/s13065-017-0340-y

**Published:** 2017-11-02

**Authors:** Antonella Leggio, Vanessa Leotta, Emilia Lucia Belsito, Maria Luisa Di Gioia, Emanuela Romio, Ilaria Santoro, Domenico Taverna, Giovanni Sindona, Angelo Liguori

**Affiliations:** 1 Dipartimento di Farmacia e Scienze della Salute e della Nutrizione, Università della Calabria, Edificio Polifunzionale, 87036 Arcavacata di Rende, CS Italy; 2 Dipartimento di Chimica e Tecnologie Chimiche, Università della Calabria, 87036 Arcavacata di Rende, CS Italy

**Keywords:** Bergamot, Essential oil, Volatile compounds, Gaseous phase, Gas chromatography–mass spectrometry, Aromatherapy

## Abstract

This work compares the composition at different temperatures of gaseous phase of bergamot essential oil at equilibrium with the liquid phase. A new GC–MS methodology to determine quantitatively the volatile aroma compounds was developed. The adopted methodology involved the direct injection of headspace gas into injection port of GC–MS system and of known amounts of the corresponding authentic volatile compounds. The methodology was validated. This study showed that gaseous phase composition is different from that of the liquid phase at equilibrium with it.
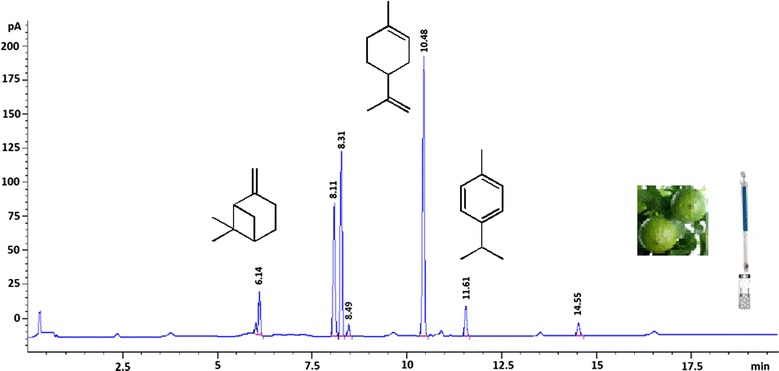

## Introduction

Phytotherapy employs fully characterized active ingredients extracted from plants for the treatment and prevention of many diseases.

Essential oils and their components exhibit various biological activities and are also used for human disease prevention and treatment. They exert antiviral, antidiabetic, antimicrobial and cancer suppressive activities [1, 2], furthermore they play a key role in cardiovascular diseases prevention including atherosclerosis and thrombosis [3, 4].

Today aromatherapy, a branch of phytotherapy, is gaining momentum as complementary therapy to the traditional medicine [5]. Aromatherapy uses essential oils via inhalation or massage as the main therapeutic agents to treat several diseases. The inhalation of volatile aromatic substances extracted from plants can affect the mood and state of health of the person by inducing psychological and physical effects [6–10]. The transdermal and transmucosal application of essential oils also concerns the phytotherapy [11].

Recently, some papers [12, 13] have tried to give scientific value to the aromatherapy, traditionally based on empirical observations and evaluations also poorly stringent, by establishing criteria similar to those that support the rigorous scientific research [14]. It has been verified in fact, which among hundreds of papers related to aromatherapy inhalation only a few are scientifically significant [15].

In order to use the essential oil appropriately it is important knowing its chemical compositions and characteristics. It seems clear, however, that if the essential oils are delivered by inhalation, the determination of gas phase (or headspace) composition above the liquid essential oil sample becomes critical [16, 17].

The migration of volatile molecules into the headspace phase does not just depend on their volatility but also on their affinity for the liquid phase sample; volatile compounds relative concentrations between the two phases will reach an equilibrium value. At equilibrium, the partial pressure of each volatile component in the headspace vapor will be equivalent to the vapor pressure that is directly proportional to its mole fraction in the liquid phase. In essence, the concentration of a compound in the headspace is proportional to its concentration in the liquid phase and can be affected by temperature, respective volumes of the sample and the headspace, and other factors [18]. Thus, headspace phase composition can be very different from that of the liquid phase.

Over the years specific studies designed to identify an analysis procedure for the determination of headspace gas at equilibrium with liquid essential oil have been reported [19–21]. These works are mostly based on the use of solid-phase microextraction (SPME) by which the headspace gas is extracted by a fused silica fiber coated with a suitable stationary phase (HS-SPME) [19, 20]. The volatile compounds adsorbed on the fiber are then thermally desorbed in the GC injector port of a GC–MS instrument to perform the qualitative analysis and GC–FID for the quantitative determination [22].

However, the composition of volatile compounds adsorbed on the fiber is different from that of headspace gas in equilibrium with the essential oil since the adsorption on the fiber depends on the fiber characteristics and extraction conditions used for the analysis. Therefore, this procedure is not sufficient to define the actual composition of the vapor phase in equilibrium with the essential oil, and hence poorly applicable to the study of aromatherapy.

Bergamot (*Citrus bergamia*) is an endemic plant of the Calabria region in the south of Italy and its fruit is used for the extraction of bergamot essential oil (BEO). Bergamot essential oil is the basic component of perfumes and is also used in the formulation of cosmetic products, food and confections as a flavouring.

The therapeutical applications of Bergamot essential oil are related to its antiseptic, antibacterial and anti-inflammatory properties. Of particular interest is also the composition of bergamot juice and albedo because of the presence of molecules with important biological and pharmacological activities [23–26]. Furthermore, it is employed in aromatherapy as an antidepressant to reduce anxiety and stress by improving mood and facilitating sleep induction [27–33].

The determination of headspace composition in bergamot essential oil is extremely useful in aromatherapy. Nevertheless, greater efforts are still needed to develop a simple and objective methodology.

In the present work, we studied the composition of the gaseous phase at equilibrium with the liquid phase of bergamot essential oil by developing a gas chromatography–mass spectrometry (GC–MS) method useful for the determination of the volatile aroma components.

## Experimental

### Materials

Bergamot essential oil (*Citrus bergamia* Risso et Poiteau) was supplied by the “Consorzio del Bergamotto di Reggio Calabria” (Southern Italy).

### Chemicals and reagents

α-Pinene, α-fellandrene, α-terpinene, linalyl acetate, neral, geranial were purchased from Sigma-Aldrich Co. (Italy). β-Pinene, *p*-cimene, γ-terpinene, terpinolene, linalool, α-terpineol were purchased from Fluka. Mircene, ocimene, neryl acetate, octyl acetate, β-caryophyllene and limonene were purchased from Merck KGaA. Anisole was purchased from Sigma-Aldrich Co (Italy) and used as internal standard.

### GC–MS analysis

GC–MS analyses were performed using a 6890N Network GC System (Agilent Technologies Inc., Palo Alto, CA) equipped with a HP-35MS (35% diphenylsiloxane; l = 20 m, d = 0.25 mm 0.25 µm) capillary column and with a mass spectrometer 5973 Network MSD operated in electron impact ionization mode (70 eV). GC–MS analyses were carried out in split mode, using helium as the carrier gas (1 mL/min flow rate). The column was maintained at an initial temperature of 40 °C for 0 min, then ramped to 250 °C at 3 °C/min, to 280 °C at 5 °C/min, where it was maintained for 15 min. Quantitative GC–MS analysis was carried out in splitless mode (splitless time, 1 min), by using anisole as the internal standard. The identification of the compounds was based on comparison of their retention times with those of authentic samples, and on comparison of their EI-mass spectra with the NIST/NBS, Wiley library spectra and literature [26]. 

### GC–FID analysis

GC–MS analyses were performed using a HP6890 A series 2 GC System (Agilent Technologies Inc., Palo Alto, CA) equipped with a HP-35MS (35% diphenylsiloxane; I = 20 m, d = 0.25 mm 0.25 µm). The column temperature was programmed at 40 °C for 0 min, to 250 °C at 3 °C/min, to 280 °C at 5 °C/min, where it was maintained for 15 min. The injector and detector temperatures were programmed at 230 and 300 °C, respectively. Helium was used as the carrier gas at a flow rate 1 mL/min.

### Quantitative analysis of bergamot essential oil

#### Sample preparation

Three aliquots of the essential oil bergamot (55, 95 and 147 mg), containing anisole (0.1 mL) as internal standard, were diluted to 5 mL with diethyl ether and then subjected to the quantitative analysis. Quantitative data were obtained by comparing the analyte/anisole area ratios in the standard solutions with the corresponding ratios in the oil samples solutions.

#### Internal standard solution

40 mg of anisole were diluted to 100 mL with diethyl ether.

#### Preparation of stock solutions A–D

For the quantitative analysis of β-pinene limonene, γ-terpinene, linalool, linalyl acetate, five stock solutions A were prepared using 150 mg of each analytes and dissolving them in 5 mL of diethyl ether. Solutions A were further used to prepare diluted working solutions B. In particular, 0.1, 0.2, 0.5, 1, 1.3 and 1.5 mL of each stock solution A, after adding 0.1 mL of the internal standard solution, was made up to 5 mL volume with diethyl ether. The final concentrations of each analyte in working solutions B were 0.6, 1.2, 3, 6, 7.8, 9.6 mg/mL respectively.

For the quantitative analysis of α-pinene, α-phellandrene, α-terpinene, p-cimene, terpinolene, myrcene, ocimene, neral, geranial, neryl acetate, α-terpineol, octyl acetate, caryophyllene, thirteen stock solutions C were prepared as follows: 50 mg of each analyte was diluted to 100 mL with diethyl ether. Aliquots of these solutions C were then used to prepare diluted working solutions D. In particular, 0.2, 0.5, 1, 1.3, 1.7 and 2.5 mL of each analyte, after adding 0.1 mL of the internal standard solution, was made up to 5 mL volume with diethyl ether. The final concentrations of each analyte in working solutions D were 0.02, 0.05, 0.10, 0.13, 0.17, 0.21 mg/mL.

### Quantitative analysis of the gaseous phase of bergamot essential oil

#### Sample preparation

Three samples of the gaseous phase of the bergamot essential oil were prepared as follows: 100 mg of bergamot essential oil and 7 mg of anisole used as the internal standard, were transferred to three 10 mL vials that were sealed and then maintained at 0, 22 and 40 °C respectively.

The temperature of 0 °C was obtained using an ice bath in which liquid phase and solid phase coexist. The temperature of 22 °C was that measured in a conditioned environment at 22 °C. 40 °C was obtained by means of a thermostated oil bath with a digital vertex thermometer.

After 30 min, a gastight syringe was used to weigh out the gaseous phase (0.4 mL) and then subjected to the quantitative analysis by both GC–MS and GC–FID. Quantitative data were obtained by comparing the analyte/anisole area ratios in the standard solutions with the corresponding ratios in the essential oil samples solutions.

#### Internal standard solution

20 mg of anisole was diluted to 500 mL with diethyl ether.

### Preparation of stock solutions for the quantitative analysis at 0 °C (Table 1)

#### Preparation of stock solutions E–H

For the quantitative analysis of α-pinene, p-cimene, mircene, linalool, linalyl acetate at 0 °C, five stock solutions E were prepared using 50 mg of each analytes and dissolving them in 100 mL of diethyl ether. Solutions E were further used to prepare diluted working solutions F. In particular, 0.3, 0.5, 1, 1.3, 1.5, 1.7 mL of each stock solution E, after adding 0.1 mL of the internal standard solution, was made up to 10 mL volume with diethyl ether. The final concentrations of each analyte in working solutions F were 0.015, 0.025, 0.050, 0.065, 0.075 and 0.085 mg/mL respectively.

**Table 1:** Stock solutions for the quantitative analysis at 0 °C

Stock solutions F α-Pinene*; p*-cimene; mircene; linalool	Concentration for each analyte (mg/mL)
Solution 1	0.015
Solution 2	0.025
Solution 3	0.050
Solution 4	0.065
Solution 5	0.075
Solution 6	0.085

Stock solutions H Limonene; β-pinene	Concentration for each analyte (mg/mL)
Solution 1	0.225
Solution 2	0.30
Solution 3	0.375
Solution 4	0.450
Solution 5	0.525
Solution 6	0.60

For the quantitative analysis of limonene and β-pinene at 0 °C, two stock solutions G were prepared using 150 mg of each analytes and dissolving them in 100 mL of diethyl ether. Solutions G were further used to prepare diluted working solutions H. In particular, 1.5, 2, 2.5, 3, 3.5 and 4 mL of each stock solution G, after adding 0.1 mL of the internal standard solution, was made up to 10 mL volume with diethyl ether. The final concentration of each analyte in working solutions H were 0.225, 0.30, 0.375, 0.450, 0.525 and 0.60 mg/mL respectively.

### Preparation of stock solutions for the quantitative analysis at 22 °C (Table 2)

#### Preparation of stock solutions I–N

For the quantitative analysis of α-phellandrene, α-terpinene, *p*-cimene, mircene, linalyl acetate at 22 °C, five stock solutions I were prepared using 10 mg of each analytes and dissolving them in 100 mL of diethyl ether. Solutions I were further used to prepare diluted working solutions J. In particular, 0.2, 0.4, 0.6, 0.8, 1.0, 1.5 mL of each stock solution I, after adding 0.1 mL of the internal standard solution, was made up to 10 mL volume with diethyl ether. The final concentrations of each analyte in working solutions J were 0.002, 0.004, 0.006, 0.008, 0.010 and 0.015 mg/mL respectively.

**Table 2:** Stock solutions for the quantitative analysis at 22 and 40 °C

Quantitative analysis at 22 °C		Quantitative analysis at 40 °C	
Stock solutions J α-Phellandrene; α-terpinene; *p*-cimene; mircene; linalyl acetate	Concentration or each analyte (mg/mL)	Stock solutions P α-Terpinene*; p*-cimene; mircene;	Concentration for each analyte (mg/mL)
Solution 1	0.002	Solution 1	0.001
Solution 2	0.004	Solution 2	0.002
Solution 3	0.006	Solution 3	0.004
Solution 4	0.008	Solution 4	0.006
Solution 5	0.010	Solution 5	0.008
Solution 6	0.015	Solution 6	0.010

Quantitative analysis at 22 °C		Quantitative analysis at 40 °C	
Stock solutions L α-Pinene; γ-terpinene; linalool	Concentration for each analyte (mg/mL)	Stock solutions R Octyl acetate; α-phellandrene; α-pinene	Concentration for each analyte (mg/mL)
Solution 1	0.050	Solution 1	0.010
Solution 2	0.065	Solution 2	0.015
Solution 3	0.085	Solution 3	0.020
Solution 4	0.10	Solution 4	0.025
Solution 5	0.120	Solution 5	0.030
Solution 6	0.140	Solution 6	0.035

Quantitative analysis at 22 °C		Quantitative analysis at 40 °C	
Stock solutions N Limonene; β-pinene	Concentration for each analyte (mg/mL)	Stock solutions T Limonene; β-pinene; linalyl acetate; γ-terpinene; linalool	Concentration for each analyte (mg/mL)
Solution 1	0.10	Solution 1	0.070
Solution 2	0.20	Solution 2	0.150
Solution 3	0.30	Solution 3	0.20
Solution 4	0.40	Solution 4	0.250
Solution 5	0.50	Solution 5	0.30
Solution 6	0.60	Solution 6	0.350

For the quantitative analysis of α-pinene, γ-terpinene and linalool at 22 °C, three stock solutions K were prepared using 50 mg of each analytes and dissolving them in 100 mL of diethyl ether. Solutions K were further used to prepare diluted working solutions L. In particular, 1.0, 1.3, 1.7, 2.0, 2.4, 2.8 mL of each stock solution K, after adding 0.1 mL of the internal standard solution, was made up to 10 mL volume with diethyl ether. The final concentrations of each analyte in working solutions L were 0.05, 0.065, 0.085, 0.10, 0.12, 0.14 mg/mL respectively. For the quantitative analysis of limonene and β-pinene at 22 °C, two stock solutions M were prepared using 100 mg of each analytes and dissolving them in 100 mL of diethyl ether. Solutions M were further used to prepare diluted working solutions N. In particular, 1.0, 2.0, 3.0, 4.0, 5.0 and 6.0 mL of each stock solution M, after adding 0.1 mL of the internal standard solution, was made up to 10 mL volume with diethyl ether. The final concentrations of each analyte in working solutions N were 0.10, 0.20, 0.30, 0.40, 0.50 and 0.60 mg/mL respectively.

### Preparation of stock solutions for the quantitative analysis at 40 °C (Table 2)

#### Preparation of stock solutions O–T

For the quantitative analysis of α-terpinene, *p*-cimene and mircene, at 40 °C, three stock solutions O were prepared using 10 mg of each analytes and dissolving them in 100 mL of diethyl ether. Solutions O were further used to prepare diluted working solutions P. In particular, 0.1, 0.2, 0.4, 0.6, 0.8, 1.0 mL of each stock solution O, after adding 0.1 mL of the internal standard solution, was made up to 10 mL volume with diethyl ether. The final concentrations of each analyte in working solutions P were 0.001, 0.002, 0.004, 0.006, 0.008 and 0.01 mg/mL respectively. For the quantitative analysis of α-pinene, α-phellandrene and octylacetate, at 40 °C, two stock solutions Q were prepared using 10 mg of each analytes and dissolving them in 100 mL of diethyl ether. Solutions Q were further used to prepare diluted working solutions R. In particular, 1.0, 1.5, 2.0, 2.5, 3.0, 3.5 mL of each stock solution Q, after adding 0.1 mL of the internal standard solution, was made up to 10 mL volume with diethyl ether. The final concentrations of each analyte in working solutions R were 0.010, 0.015, 0.020, 0.025, 0.030, 0.035 mg/mL respectively. For the quantitative analysis of limonene, β-pinene, linalyl acetate, γ-terpinene and linalool at 40 °C, five stock solutions S were prepared using 100 mg of each analytes and dissolving them in 100 mL of diethyl ether. Solutions S were further used to prepare diluted working solutions T. In particular, 0.7, 1.5, 2.0, 2.5, 3.0 and 3.5 mL of each stock solution S, after adding 0.1 mL of the internal standard solution, was made up to 10 mL volume with diethyl ether. The final concentrations of each analyte in working solutions T were 0.07, 0.15, 0.20, 0.25, 0.30 and 0.35 mg/mL respectively.

### Statistical analysis

Statistical analyses were carried out with the SPSS Statistics 23.0 (SPSS Inc., Chicago, IL, USA). For each compound, six solutions were prepared and analyzed by GC–MS. The statistical analysis was obtained comparing the analyte/anisole area ratios in the solutions with the corresponding concentrations. A value of P correspondent to 0.011 was considered significant.

## Results and discussion

The distilled bergamot essential oil used was preliminarily analyzed to define its composition. The individual analytes present in the oil were identified by GC–MS methodology by comparing the corresponding retention times and mass spectra with those of authentic sample (Table 3).

**Table 3:** Composition of BEO and gaseous phase in equilibrium with the liquid at 0 °C

Entry	Compound	Essential oil composition^a^		Gaseous phase composition at 0 °C		
		t_R_ (GC/MS) (min)	GC–MS (w/w% ± SD)	GC–MS (w/w% ± SD)	GC–FID (w/w% ± SD)	t_R_ (GC/FID) (min)
*Cyclic hydrocarbon monoterpenes*						
1	α-Pinene	6.14	1.03 ± 0.10	6.90 ± 0.10	7.06 ± 0.25	6.14
2	β-Pinene	8.19	6.56 ± 0.14	25.90 ± 0.40	26.68 ± 0.15	8.31
3	α-Phellandrene	9.48	0.04 ± 0.01	–	–	–
4	α-Terpinene	9.94	0.16 ± 0.02	–	–	–
5	Limonene	10.60	30.20 ± 0.77	58.07 ± 0.38	57.12 ± 0.35	10.48
6	*p*-Cimene	11.19	0.18 ± 0.01	6.36 ± 0.04	6.02 ± 0.08	11.61
7	γ-Terpinene	12.15	11.95 ± 0.32	–	–	–
8	Terpinolene	13.36	0.27 ± 0.03	–	–	–
*Acyclic hydrocarbon monoterpenes*						
9	Mircene	8.72	0.82 ± 0.02	2.19 ± 0.22	2.16 ± 0.15	8.49
10	Ocimene	11.03	0.08 ± 0.01	–	–	–
*Acyclic oxygenated hydrocarbon monoterpenes*						
11	Linalool	14.58	21.82 ± 0.87	3.04 ± 0.54	2.96 ± 0.33	14.55
12	Linalyl acetate	21.42	16.21 ± 0.84	–	–	–
13	Neral	22.94	0.21 ± 0.01	–	–	–
14	Geranial	24.46	0.11 ± 0.01	–	–	–
15	Neryl acetate	28.14	0.28 ± 0.02	–	–	–
*Cyclic oxygenated hydrocarbon monoterpenes*						
16	α-Terpineol	20.01	0.87 ± 0.08	–	–	–
*Esters*						
17	Octyl acetate	19.63	0.10 ± 0.01	–	–	–
*Sesquiterpenes*						
18	β-Caryophyllene	27.85	0.14 ± 0.02	–	–	–

*SD* standard deviations

^a^The w/w percentages were determined by the internal standard method and referred to the amount of each component contained in 100 g of essential oil

Anisole was chosen as internal standard for the quantitative measurement of the individual analytes. 

For the quantitative analysis, six standard stock solution (Stock B and Stock C) containing different concentration levels of each identified analyte and the same amount of internal standard were prepared.

Each solution was injected in triplicate in the GC–MS system under optimized conditions. For each measurement, the concentration and the peak area of the analytes were compared with those of the internal standard.

Table 1 reports the quantitative results only for the identified analytes.

High contents of limonene, linalool, linalyl acetate, and α-terpinene are observed in analogy with the data reported in literature [18, 33, 34].

The determination of gas phase composition above the liquid oil has preliminarily required controlled temperature and pre-established equilibrium conditions.

**Fig. 1:**
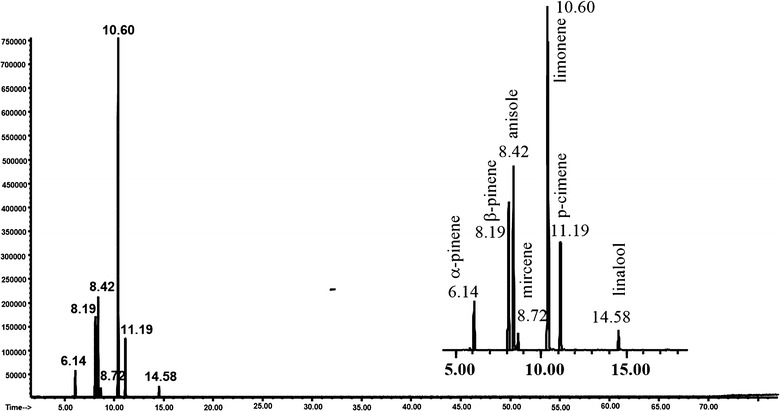
GC–MS analysis of the gaseous phase of bergamot essential oil at 0 °C (α-pinene t_R_ = 6.14 min; β-pinene t_R_ = 8.19 min; anisole t_R_ = 8.42 min; mircene t_R_ = 8.72 min; limonene t_R_ = 10.60 min; *p*-cimene t_R_ = 11.19 min; linalool t_R_ = 14.58 min)

To this aim, a weighed amount of essential oil was placed in a headspace vial, after adding a given amount of anisole the vial was sealed and then allowed to stand for 30 min at 0 °C to establish the equilibrium at that temperature. Once the volatile compounds have equilibrated, an aliquot of the headspace gas was withdrawn using a gas tight syringe, injected into the gas chromatograph injection port and analyzed by GC–MS. The individual analytes present in the headspace gas were identified through comparison of retention times and mass spectral data with those of authentic standards (Fig. 1).

Additional experiments using equilibration times longer than 30 min were also carried out. After 60 min equilibration time the relative ratios between the different volatile components did not change significantly compared to those obtained after 30 min.

For the quantitative analysis, seven stock solutions containing the reference analytes at known concentrations and a given amount of anisole as internal standard were used. An aliquot (1 µL) of each of these stock solutions (Stock F and Stock H) was injected into the GC–MS injection port where it was completely turned to gas and analyzed. All the analyses were performed in splitless conditions in triplicate.

The determination of each analyte concentration level in the headspace gas of essential oil sample was performed by comparing the peak area of each individual headspace analyte with the corresponding peak area in the reference solutions, the peak area of the analytes are always compared with those of the internal standard.

The adopted methodology assumes that the total sample amount introduced into the injection port is vaporized and that all the produced gas reaches the ion source (splitless conditions).

The quantitative results are listed in Table 3.

**Fig. 2:**
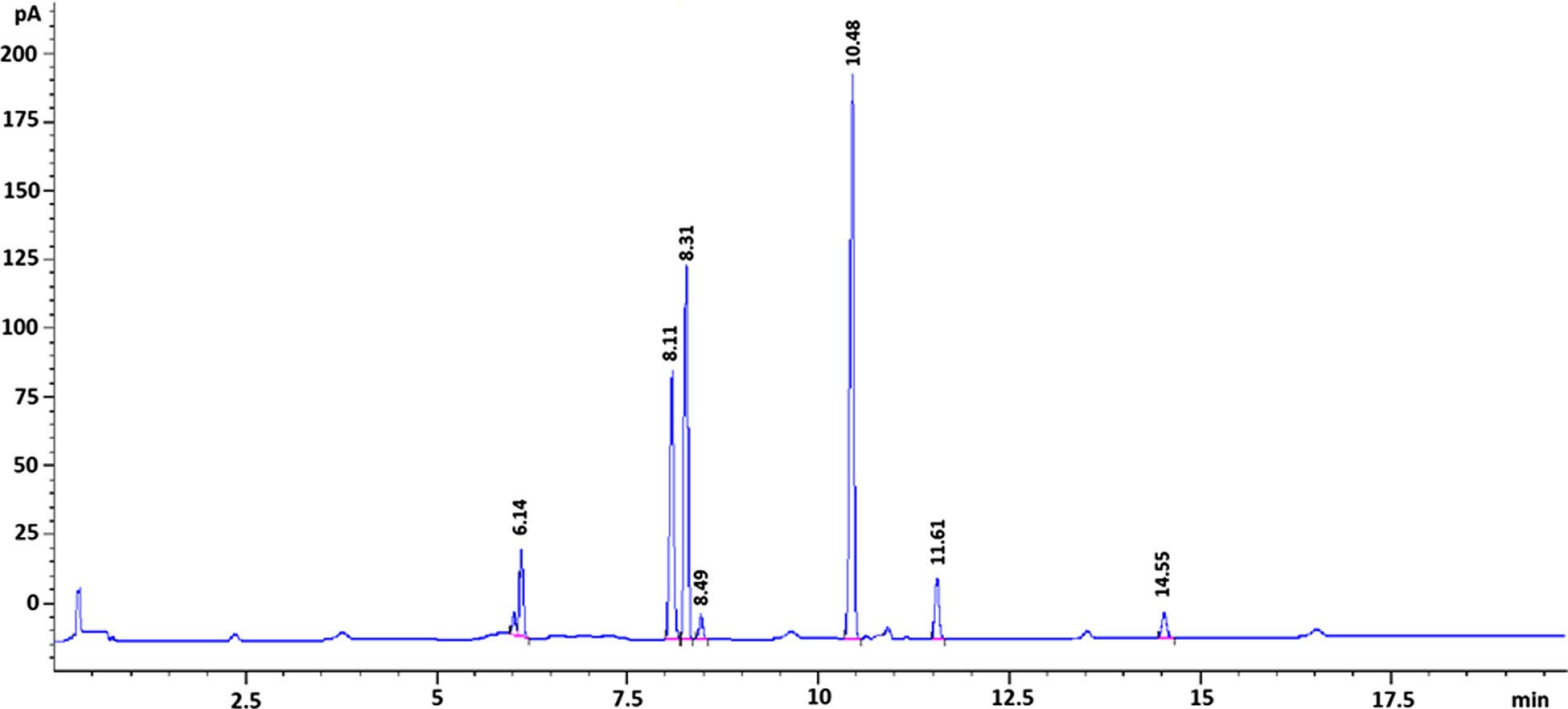
GC–FID analysis of the gaseous phase of bergamot essential oil at 0 °C (α-pinene t_R_ = 6.14 min; β-pinene t_R_ = 8.11 min; anisole t_R_ = 8.31 min; mircene t_R_ = 8.49 min; limonene t_R_ = 10.48 min; *p*-cimene t_R_ = 11.61 min; linalool t_R_ = 14.55 min)

In this study, the headspace gas in equilibrium with the bergamot oil sample at 0 °C has been also investigated by means of GC–FID in order to validate the proposed methodology (Fig. 2).

The results of GC–FID analysis are comparable to those obtained by GC–MS (Table 3). It can be observed that the gaseous phase composition is quite different from that of the liquid phase at equilibrium with it a 0 °C.

The comparison between the bergamot essential oil composition (Table 3) and that of headspace gas at equilibrium shows how the linalool and the linalyl acetate amounts decrease dramatically in the gas phase on the contrary the concentration of limonene is almost double (approximately 60%).

Furthermore, the β-pinene content, that is very low in the liquid oil, is particularly high in gaseous phase.

The composition of the gaseous phase at 22 °C (room temperature) and 40 °C was determined by using the stock solutions *I*–*N* and *O*–*T* respectively as described in “Experimental” section. The quantitative results are listed in Table 4.

**Table 4:** Composition of BEO and gaseous phase in equilibrium with the liquid at 0, 22 and 40 °C

Entry	Compound	Essential oil composition^a^	Gaseous phase composition at 0 °C	Gaseous phase composition at 22 °C	Gaseous phase composition at 40 °C	Biological activity
		GC–MS (w/w% ± SD)	GC–MS (w/w% ± SD)	GC–MS (w/w% ± SD)	GC–MS (w/w% ± SD)	
*Cyclic hydrocarbon monoterpenes*						
1	α-Pinene	1.03 ± 0.10	6.90 ± 0.10	5.38 ± 0.10	1.29 ± 0.03	Anticancer [35] Anti-inflammatory [36]
2	β-Pinene	6.56 ± 0.14	25.90 ± 0.40	19.69 ± 0.31	7.10 ± 0.05	Anti-depressant [37] Antibacterial [38]
3	α-Phellandrene	0.04 ± 0.01	–	0.27 ± 0.02	0.39 ± 0.02	Anti-proliferative [39] Anti-inflammatory [40]
4	α-Terpinene	0.16 ± 0.02	–	0.27 ± 0.01	0.18 ± 0.02	Antioxidant [41]
5	Limonene	30.20 ± 0.77	58.07 ± 0.38	47.27 ± 0.28	37.15 ± 0.29	Anti-inflammatory [42, 43] Anxiolytic [44] Anti-proliferative [45, 46]
6	*p*-Cimene	0.18 ± 0.01	6.36 ± 0.04	0.62 ± 0.03	0.49 ± 0.01	Anti-inflammatory [47] Antifungal [48]
7	γ-Terpinene	11.95 ± 0.32	–	13.13 ± 0.29	12.22 ± 0.1	Antibacterial [49] Antioxidant [49]
8	Terpinolene	0.27 ± 0.03	–	–	–	
*Acyclic hydrocarbon monoterpenes*						
9	Mircene	0.82 ± 0.02	2.19 ± 0.22	1.42 ± 0.036	0.84 ± 0.02	Analgesic [50] Anxiolytic [51, 52]
10	Ocimene	0.08 ± 0.01	–	–	–	
*Acyclic oxygenated hydrocarbon monoterpenes*						
11	Linalool	21.82 ± 0.87	3.04 ± 0.54	9.71 ± 0.18	27.52 ± 0.24	Anti-inflammatory [53, 54] Anti-epileptic [55] Anxiolytic [56]
12	Linalyl acetate	16.21 ± 0.84	–	0.66 ± 0.03	10.40 ± 0.08	Anti-inflammatory [57] Analgesic [57] Antibacterial [58]
13	Neral	0.21 ± 0.01	–	–	–	
14	Geranial	0.11 ± 0.01	–	–	–	
15	Neryl acetate	0.28 ± 0.02	–	–	–	
*Cyclic oxygenated hydrocarbon monoterpenes*						
16	α-Terpineol	0.87 ± 0.08	–	–	–	
*Esters*						
17	Octyl acetate	0,10 ± 0.01	–	–	1.91 ± 0.04	Anti-inflammatory [59] Analgesic [59]
*Sesquiterpenes*						
18	β-Caryophyllene	0.14 ± 0.02	–	–	–	

*SD* standard deviations

^a^The w/w percentages were determined by the internal standard method and referred to the amount of each component contained in 100 g of essential oil

At 22 °C the gas phase in equilibrium with liquid phase is enriched in some components with respect to the composition determined at 0 °C. In fact, α-phellandrene, α-terpinene, γ-terpinene, linalool and linalyl acetate, which were not detected in the gaseous phase at 0 °C, were identified and determined in the gaseous phase at 22 °C. In particular, at 22 °C γ-terpinene and linalyl acetate got to 13.13 and 0.66% respectively and linalool grew from 3 to 9% (Table 2). At both temperature, the main components were limonene (58.07% at 0 °C and 47.27% at 22 °C) and β-pinene (25.90% at 0 °C and 19.69% at 22 °C).

The composition of the headspace vapor generated at 40 °C was characterized by the presence of octyl acetate, not detected at 22 °C, and the significant decrease of limonene, and α and β-pinene. On the contrary, linalool and linalyl acetate were appreciably increased contributing to the composition of the gaseous phase of BEO at 40 °C with 27.52 and 10.40%, respectively (Table 4).

By comparing the composition of bergamot essential oil with those of the gaseous phase in equilibrium with the liquid phase of BEO at 0, 22 and 40 °C, we observed that seven components of bergamot essential oil (terpinolene, ocimene, neral, gerianal, neryl acetate, α-terpineol, β-cariofyllene) were totally absent in the compositions of all analyzed gaseous phases.

Additionally both in the essential oil and gaseous phase at 40 °C the major components, albeit with different percentages, are limonene, linalool, γ-terpinene and linalyl acetate (Table 4).

All these results showed that the compositions of the gaseous phases of BEO generated at various temperatures (0, 22 and 40 °C) are different and change also respect to the composition of the essential oil. Many of the components present in the essential oil are totally absent in the gas phase even at 40 °C while others, present in small portion in the essential oil, are concentrated in the gaseous phase.

The model we studied represents a closed system that, with some limits, mimics the open system in which aromatherapy is usually performed where the gas composition should change until the equilibrium is achieved in the room environment.

Therefore our system could approximate the conditions under which aromatherapy is practiced.

## Conclusion

These results suggest that the determination of the gaseous phase composition in equilibrium with the liquid essential oil is critical for establishing the correlation between the volatile components and their activity.

This study showed that for employing bergamot essential oil in aromatherapy it is not enough to know the essential oil composition but is extremely important to know the volatile fraction composition in equilibrium with it.

This paper reports a GC–MS methodology for the direct analysis of volatile compounds of bergamot essential oil.

The method can also be applied to environments of greater volume provided that the parameters relating to temperature are maintained and that there exist conditions whereby the vapor phase is in equilibrium with the essential oil.

The developed method is quite general and can be applied to other vegetable matrices.
